# Infaunal Benthic Communities from the Inner Shelf off Southwestern Africa Are Characterised by Generalist Species

**DOI:** 10.1371/journal.pone.0143637

**Published:** 2015-11-30

**Authors:** Nina Steffani, Safiyya Sedick, John Rogers, Mark John Gibbons

**Affiliations:** 1 21 Skippers End, Zeekoevlei, Cape Town, South Africa; 2 Department of Biodiversity and Conservation Biology, University of the Western Cape, Bellville, South Africa; 3 41 Newlands Avenue, Newlands, Cape Town, South Africa; Università di Genova, ITALY

## Abstract

Infaunal communities of benthic macro-organisms (≥ 1mm length) were studied from 81 samples collected across nine sites to the north and south of the Orange River in the Benguela upwelling ecosystem in 2003, with a view to describing communities and understanding the drivers of regional community structure, as well as to document diversity and to examine geographic affinities. Although the fauna was dominated by polychaetes and peracarid crustaceans, patterns in community structure could only weakly be explained by the measured environment (~35%). This is attributed to the generalist nature of the species recovered, which were widely distributed amongst different sediments, water-depths and latitudes. The fauna is dominated by species that enjoy a widespread regional and global distribution and is characterised by relatively low diversity, which is discussed.

## Introduction

Coastal-upwelling ecosystems are amongst the most productive in the world, where pelagic systems are characterised by short diatom-based food chains leading to industrial scale fisheries [1]. Biomass is generally high, and diversity is correspondingly low, perhaps because the high levels of environmental instability prevents the fine-tuning of genotypes to local conditions [2]. Although this favours habitat generalists (as at polar latitudes [3]), few of the dominant species are shared amongst the four (Benguela, Humboldt, Canary and California) major upwelling systems [2]: and those that are tend to be large and migratory.

The benthic habitat of these upwelling ecosystems is often characterised by large areas of diatomaceous ooze, fed by the sedimentation of excess surface production [4]. This environment is associated with low concentrations of dissolved oxygen (<0.5 ml O_2_ l^-1^), and sometimes high levels of hydrogen sulphide in the pore water [5]. Methane eruptions are not uncommon [6]. The fauna of these oxygen minimum zones (OMZs), *sensu* [7], is characterised by a high density of benthic Foraminifera, which is thought to reflect both their release from predation and an enhanced food supply [7]. The densities of macrofauna (>1 mm body length) are typically low, except at the margins, and communities tend to be dominated by polychaete worms of small size [7 *cf* 8]. Macrofaunal diversity within OMZs is also low, and communities show a remarkable homogeneity in composition across wide spatial scales; a fact that is attributed to the lack of biogenic structures and large burrowers [7]. Although the typical fauna of OMZs has a variety of specialised adaptations to low oxygen [7], periodic increases in oxygenation can lead to the sudden proliferation of a diversity of previously absent, highly opportunistic species such as molluscs and crustaceans, as well as echinoderms [9].

Despite the presence of permanent OMZs in coastal upwelling areas, these systems often support important demersal fisheries [10]. And whilst some of the target species are piscivorous as adults (e.g. hakes), most will feed on benthic infaunal organisms for at least part of their life cycle (e.g. [11]). Clearly, given the paucity of animals in the permanent OMZs, these areas cannot provide all the necessary food for the often abundant demersal resources (but see [12]), yet our knowledge of benthic communities from outside of the OMZs in upwelling areas is curiously limited.

In the case of the Benguela upwelling ecosystem, macrofaunal diversity appears to be lowest off Walvis Bay in Namibia [8], which is attributed to the OMZ that is perennially present over the shelf there [13]. Off the Kunene River in the north, diversity has been observed to increase significantly [8, 14–15] and this reflects both biogeographic influences (higher natural diversity in the subtropical waters off southern Angola) and a movement away from Walvis Bay. An increase in diversity has been noted too by Atkinson et al [16] to the south of Walvis Bay, which may also be related to the more oxic nature of the overlying water column (>2 ml O_2_ l^-1^), but could also be due to the increased water-depth (348–436 m cf 49–117 m, [8]) from which they collected their samples.

The factors contributing to the diversity of local infaunal communities outside of OMZs have been reviewed by [17], but it is widely agreed that the regional species pool, which reflects bathymetry and latitude, is primarily responsible for influencing local diversity. Gray ([17] pp 293) further suggests that “…available food resources control population densities at a variety of scales and set the maximum range of species richness, but that variability in species richness for a given resource level is determined by spatial and temporal heterogeneity in sediment structure…”. However, the few regional studies that have attempted to look at infaunal communities have indicated that sediment properties explain either no (e.g. [8]) or a limited [16] amount of the variability in communities or their attributes.

A reduction in diversity around the coast of South Africa from east to west has been widely noted in the literature for intertidal and shallow water communities and taxa (e.g. [18]). Although patterns of endemism vary with the subject group, most taxa show greatest levels of endemism along the south coast (e.g. ascidians), and at the transition areas between regional biogeographic provinces [19–20]. The west coast region supports low numbers of both overall species and endemic species [19]. Few benthic studies have extended into Namibia (e.g. [18]), but the results of work on pelagic organisms suggest a further decline in diversity to the position of the Benguela-Angola Front [21].

Here, we set out to examine infaunal communities from the offshore (inner shelf only) waters of southern Namibia (outside the permanent OMZ at Walvis Bay) and off the northwest coast of South Africa (Namaqualand) with a view to 1) describing and exploring spatial patterns in community structure and the potential drivers of same, 2) estimating species richness for selected taxa and so further our insight into regional patterns of diversity, and 3) examining the global distributional ranges of the fauna in order to establish biogeographic affinities and levels of endemism.

## Materials and Methods

### Study Area

**Fig 1:**
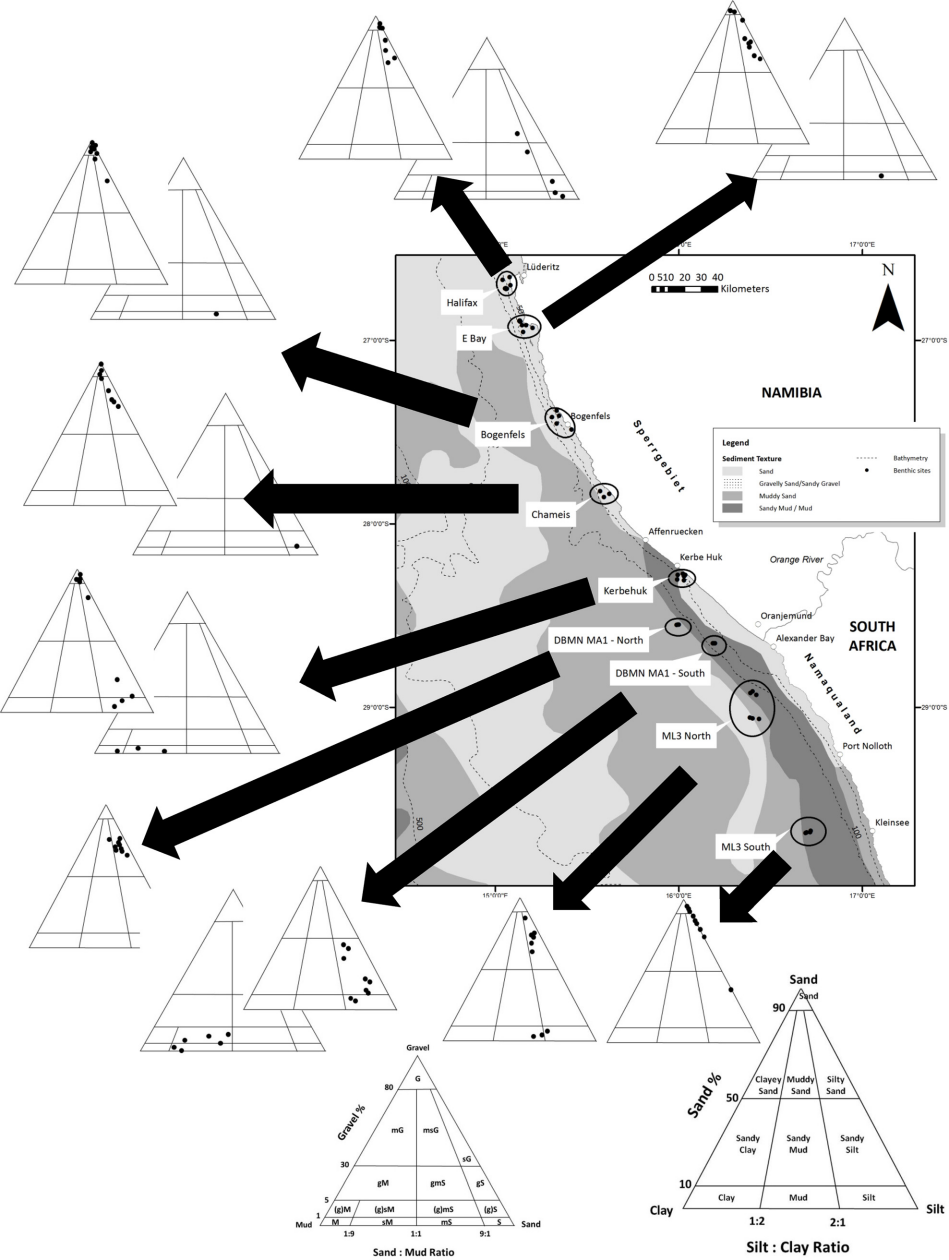
Map of sampling region. The position of the different samples collected from the nine sites during 2003 (made using ArcGIS, Version 10.2 using data from [26]). Place names mentioned in the text, and bathymetry (m) also shown. Gravel-sand-mud, and mud-silt-clay ternary diagrams for each site also shown.

The study area is located over the inner shelf north and south of the Orange River, the latter marking the political boundary between Namibia and South Africa (Fig 1). Within this area, nine sites were selected for investigation (Fig 1) The sedimentary environment of the area has been described by [22–24], whilst the physical and chemical environment have been summarised respectively by [25] and [13].

In brief, the shelf off the Orange River is up to 180 km broad [25], and sediments form textural zones parallel to the coast [22]. These generally become finer seawards, changing from terrigenous (land derived, low carbonate) sands on the inner and middle shelves to terrigenous muddy sands and sandy muds on the outer shelf and continental slope, but may remain sandy to the slope in areas where relict sand is found on the middle shelf. The extensive mud belt that stretches southwards along the South African west coast for up to 500 km is up to 40 km wide and 15 m thick and is situated over the outer edge of the inner shelf. The sediments are mainly of terrigenous origin, and on the prodelta of the Orange River they are laminated and have a dominant fluvial input [27]. The samples from ML3 South (Fig 1) come from the distal edge of the terrigenous inner-shelf mudbelt in depths of 141 m to 137 m, SW of Port Nolloth, and those from ML3 North also come from the distal edge of the inner-shelf mudbelt, WSW of the Orange River, from slightly shallower depths of 134 m to 120 m.

Off the Sperrgebiet, immediately north of the Orange River, the MA1 North and South sites (Fig 1) occur over the distal edge of the inner-shelf mudbelt, there being part of the Orange prodelta, in depths of 102 m to 98 m (South) and 107 m to 105 m (North). The gravels, initially deposited by surf-zone processes during the Pleistocene low stands in this area were drowned by quartzose sands, and then the prodeltaic silts and clays deposited by the seaward prograding-feather edge of the Holocene Orange Delta were subsequently integrated into the delta-front by bioturbation. As a consequence, the sediments here typically exhibit a fining-upward transgressive sequence within the upper 30 cm and consist mainly of muddy sand and sandy mud [23].

The five sampling-areas farther north off southern Namibia are all from shallower depths (67 m to 14 m), close to the higher-energy, wave-affected zone beside the coast. The Kerbe Huk samples, still off the Sperrgebiet, are from 67 to 22 m, those from just north of Chameis Bay are from 47 m to 21 m and those off Bogenfels from 76 m to 19 m. Approaching Lüderitz, north of the Sperrgebiet, the samples from Elizabeth Bay are from 60 m to as shallow as 14 m, whereas those off Halifax Island, just west of Lüderitz, are from 67 m to 24 m.

Much of the inner shelf off central Namibia is subject to seasonally and inter-annually variable oxygen stress [28]. This reflects the complex interplay between the sedimentation and decay of local surface production, thermal stratification and the advection of low-oxygen waters from Angola [28]. Although the Orange River Bight is subject to locally generated low-oxygen water [29], as are all areas downstream of the upwelling cells in the Benguela ecosystem [13], the impact of low-oxygen water from Angola appears limited here [30].

Companies within the De Beers Group of Companies are holders of several diamond-mining-licence areas off the South African (Namaqualand) and Namibian West Coast. The marine portions of these licence areas include the deep-sea (80–120m) mining areas MLA3 off Namaqualand and the Atlantic 1 Mining Licence (MLA1) area off the southern Namibian coast, as well as the southern Namibian mid-water (<80 m) licence areas between Kerbe Huk and Lüderitz (Fig 1).

### Benthic Sampling Methods

Nine samples were collected from each of the nine sites shown in Fig 1 during November 2003. All sites occurred within the mining-licence areas for which the respective De Beers Group companies have exclusive government permission to sample, and from whom permission was obtained to collect the material presented here. All samples were at least 1 km from the nearest mining activity. Samples were taken with a Van Veen grab, which samples an area of 0.2 m^2^ of seabed penetrating the sediment to about 20–30 cm. Although the volume of each grab sample was not explicitly determined at the time of sampling, any sample estimated to be less than 5 l in volume was discarded. Subsequent sampling (2005–2008) in the same areas has indicated that the average volume of sediment collected by the grab, as used here, is 14.21 l (95% confidence intervals 13.73 l – 14.70 l; N = 421). Given that the majority of infaunal organisms are generally located in the upper 5–10 cm of the sediment, this sample volume corresponds to an average depth of approximately 7.1 cm. Further, given that community analyses depend on a root-root transformation of abundance data (see below), any differences in organismal abundance between samples that reflects differences in sediment volume, will be minimised. That said, caution should be exercised in the interpretation of the abundance data presented. A representative wet sample of the sediment was taken for subsequent textural (granulometric) analysis. Each grab sample was wet-sieved through a 1 mm-mesh sieve and all organisms retained were fixed in a 10% buffered seawater formaldehyde solution.

### Sediment Analysis

The grain-size composition of the sediment samples taken from each grab was determined following standard ASTM D422 methods. Samples were first dried at 50°C in an oven and then passed through a 2 mm sieve. The +2 mm material was washed, dried and sieved. The – 2 mm material was dispersed with Sodium Hexametaphosphate (40g/l) (50g sediment for silt/clay samples or 100g sediment for sandy samples), hydrometer readings taken, and the suspension passed through 75 μm sieve, washed, dried and then sieved again. **S**ediment masses per sieve were expressed as percentages of the total mass recorded, according to the following Wentworth Grades: >2000 μm (Gravel), 1000–2000 μm (Very Coarse Sand), 500–1000 μm (Coarse Sand), 250–500 μm (Medium Sand), 125–250 μm (Fine Sand), 63–125 μm (Very Fine Sand) and <63 μm (Mud). The mud fraction was further subdivided into Silt (63 to 2 μm) and Clay (<2 μm) by hydrometer analysis. These sediment-fractions were further grouped into Gravel (>2000 μm), Sand (2000–63 μm) and Mud (<63 μm). To derive an indication of the mean grain size and sediment textural group (*sensu* [31]) for each sample, data were analysed using the sediment-analysis program Gradistat v8 [32] that computes grain-size statistics based on Folk and Ward [33] and the moments methods by Blott and Pye [32]. The mean water water-depth of each sediment-textural group was calculated across sites, whereas the diversity of sediment-textural groups per site was calculated using the Shannon Index [34]. Comparisons of environmental measures between sites were done using ANOVA, following data transformation (log_10_ water-depth, arcsine percentages), and Tukey post-hoc testing employed when appropriate.

The composition of the sediments was not examined under a binocular microscope, though it should be noted that gravels primarily consist of shell fragments rather than the sedimentologically more significant rock fragments.

### Faunal Analysis

The animals were identified (and counted) to the species level where possible, otherwise to genus- or family-level and data were analysed separately for each. Taxa that could be identified to genus-level, but which could not be assigned a species name as they were missing diagnostic features were not included in the species-level analyses. Similarly, taxa that could be identified to family-level, but not to genus-level (again because they lacked key features) were ignored from genus-level analyses. Planktonic species such as euphausiids or hyperiid amphipods were excluded from the analysis, as were taxa that could not be assigned as described above (e.g. cumaceans, nemerteans), or that could not be identified even to family level (e.g. decapitated polychaetes).

### Statistical Analysis: Sedimentological Data

In order to explore structure in the multivariate sedimentary environment, a similarity matrix between the data from each sample was generated based on Euclidean distance, following arcsine transformation and normalisation, using PRIMER 6 [35]. As none of the seven size-fractions were correlated with each other at a level of r >0.95, all were included in analyses (see [36]). Patterns were then investigated using Principal Components Analysis (PCA). The relationship between the texture of the sediments vs water-depth, latitude/site and distance from the Orange River mouth (as predictors, all log_10_ transformed) was investigated using a Distance Based Linear Model (DistLM) [37]. At first, DistLM conducts marginal tests, which determine the proportion of the variance in the sediment distribution pattern that can be explained by each predictor, before partitioning the variation according to a step-wise multiple regression model. The model was visualised using distance-based redundancy analysis (dbRDA), which is an ordination of the fitted values from the multivariate regression model [37].

### Statistical Analysis: Biological Data

A variety of statistical procedures in the computer package PRIMER 6 & PERMANOVA+ was used to analyse community structure (separately at species, genus and family levels) across the region, and these are described by [35–37], and other references therein. All data were first root-root transformed, and a Bray-Curtis resemblance matrix computed between samples. This was visualised using non-metric multi-dimensional scaling (MDS). Relationships between the resemblance matrices produced from studies at the species, genus and family were ascertained, pairwise, using the RELATE test in PRIMER. This is a non-parametric Mantel test, and generates a rank correlation coefficient ρ between corresponding elements from two matrices [36].

In order to determine which environmental predictors (water-depth, latitude, distance from the Orange River mouth and various sediment characteristics, including the percentages of mud, sand and gravel, as well as skewness, sorting and kurtosis) were responsible for driving any observed pattern in the biological resemblance matrix, data were analysed by a DistLM and visualised using dbRDA.

As latitude/site was identified as a key driver of community structure (see Results, below), a Similarity Percentage (SIMPER) analysis was employed to determine those species that were principally responsible for similarities between samples within a site. The SIMPER routine decomposes average Bray-Curtis similarities between all pairs of samples within a site into percentage contributions from each species [36]. The SIMPER routine was also used to identify those taxa most responsible for similarities within water-depth and sediment textural classes. Following the latter analyses, the weighted mean water-depth and sediment grain size was calculated for each taxa following standard methods.

Community attributes (number of species, genera and families as well as abundance and Shannon diversity) per sample (0.2 m^2^) were calculated. Because there were equal numbers of samples per site (nine) it was possible to compare richness across sites directly. However, as richness varies with both effort (number and size of samples) and abundance [34], and in the interests of making the data more useful to others, estimates of richness per site (and across the region) were computed using EstimateS [38]. Estimates of richness (identified species and genera) were computed only for Polychaeta and Amphipoda, because these taxa were most diverse in the samples (see Results, below) and were generally best preserved, allowing ready identification. Because the number of samples per site is too few to establish whether a species may be considered “rare”, the use of the non-parametric estimators ICE (incidence-based coverage estimator) and Chao2 [39–41] would lead to unreasonable estimates of richness estimates. We have therefore made use of the observed mean estimate as an indication of species richness. CurveExpert [42] was then used to plot the mean estimated richness value of each sample (per site) in the form of a species accumulation curve. Each curve was fitted with the Morgan- Mercer- Flodin model (MMF) whose asymptotic parameter value may be taken as the total number of species to be found within the community [43]. The MMF model was used as a means of consistency and provided a valid means of comparison between each of the accumulation curves [44].

The total number of sites and textural groups across which each species was found was determined, as were the global distributions of each. The latter were determined by searching four online databases, *viz* World Register of Marine Species (WoRMS), Ocean Biogeographic Information System (OBIS), Encyclopedia of Life (EOL) and European Register of Marine Species (ERMS). When no species records existed, the wider literature was used. Species distributions were recorded as Atlantic, Benguela (Angola, Namibia and South Africa), regional (Namibia, South Africa and Mozambique), Atlantic-Indian, global (including Antarctic and Pacific) and endemic (Namibia only).

## Results

### Sediments

There was no significant difference in water-depth among the samples collected at either the five more northern, mid-shelf sites, or the four southern offshore sites (Table 1), though the two groups were significantly different from each other (ANOVA test:F = 27.78, p<0.001; Tukey post-hoc test results in Table 1). Although sediments at most sites were dominated by sand, those collected at the two sites immediately north (MLA1 South) and south (ML3 North) of the Orange River contained large amounts of mud (Table 1). The mud content of sediments tended to decrease with increasing distance from the Orange River, and sediments were most gravelly in the north (Table 1).

**Table 1:** Summary of physical environment.

Site	Depth	% Mud	% Sand	% Gravel	H’
**Halifax**	* **56 (17.64)**	**10.44 (5.83)**	**82.57 (16.15)**	**6.99 (13.25)**	**1.43**
**Ebay**	* **36.67 (13.07)**	**18.2 (9.45)**	**81.75 (9.53)**	**0.05 (0.16)**	**0.94**
**Bogenfels**	* **41.56 (24.28)**	**5.78 (8.27)**	**94.19 (8.37)**	**0.03 (0.1)**	**0.35**
**Chameis**	* **36.67 (11.95)**	**10.69 (8.16)**	**89.08 (8.03)**	**0.24 (0.39)**	**1.1**
**Kerbehuk**	* **47.56 (19.09)**	**42.55 (42.86)**	**57.37 (42.96)**	**0.08 (0.16)**	**1.43**
**DBMN MA1 North**	**‡106 (0.87)**	**23.51 (1.61)**	**76.49 (1.61)**	**0 (0)**	**0**
**DBMN MA1 South**	**‡100 (1.73)**	**77.04 (16.07)**	**21.95 (15.08)**	**1.01 (1.29)**	**1.15**
**ML3 North**	**‡128 (5.97)**	**43.76 (38.1)**	**56.24 (38.1)**	**—-**	**1.15**
**ML3 South**	**‡139 (2.32)**	**20.15 (18.64)**	**79.85 (18.64)**	**—-**	**0.94**

Average water-depth and sediment texture encountered at each of the sites sampled off southwestern Africa during 2003: Shannon Index (H’) describing diversity of Folk’s (1954) sediment group, and sampling date also shown. Data as mean (standard deviation); N = 9, in all cases.

* Similar sampling depths, as determined from Tukey post-hoc testing, shown by common symbol.

‡ Similar sampling depths, as determined from Tukey post-hoc testing, shown by common symbol.

**Fig 2:**
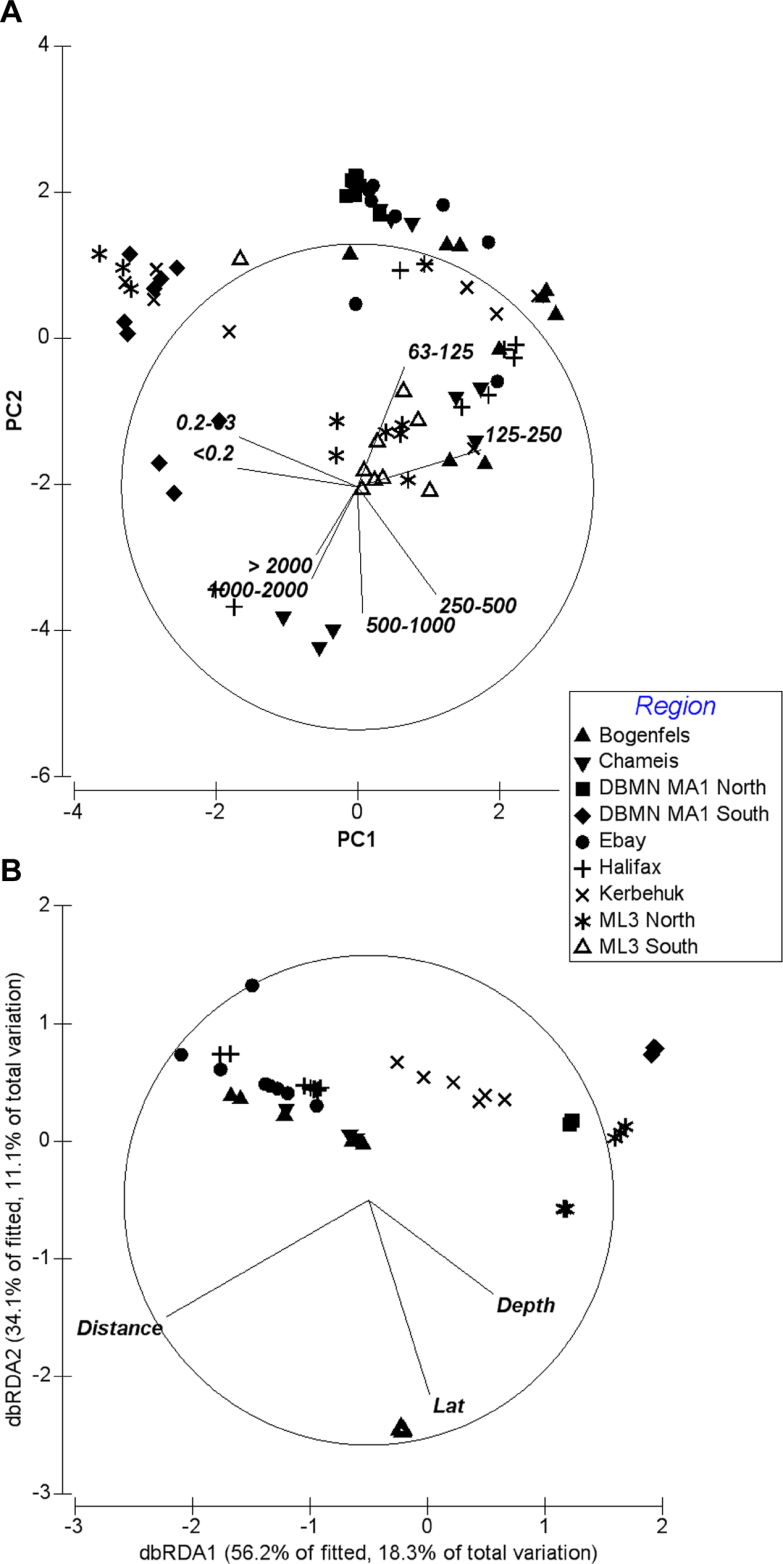
Two dimensional visualisations of sediment environment. (A) PCA plot showing the distribution of the samples collected off southwestern Africa during 2003: vectors indicate the direction of increase of the different sediment size classes. (B) Multivariate multiple regression (dbRDA) performed on sediment data and predictor variables (water-depth, latitude and distance to the Orange River mouth): vectors indicate the direction and strength of the environmental gradients. The location of the samples, by site, is indicated by symbols.

Averages mask variability, however and it is clear from Fig 1 and S1 Fig that there was quite a bit of patchiness in the sediment textures of the different samples collected within each of the different sites. That said, the results of the PCA, the first two axes of which explain about 70% of the variability in the multivariate dataset (Fig 2A), clearly separate the sediment environment across the region and the marginal tests of the DistLM indicate that distance from the Orange River mouth (pseudo-F 16.03, p = 0.001), water-depth (pseudo-F 12.30, p = 0.001) and latitude (pseudo-F 12.50, p = 0.001) were approximately equally significant as predictors of sediment texture (Fig 2B). The final model (adj R^2^ 0.299) included all predictors: distance (adj R^2^ 0.16, pseudo-F 16.03, p = 0.001), latitude (adj R^2^ 0.26, pseudo-F 11.79, p = 0.001) and depth (adj R^2^ 0.0, pseudo-F 5.45, p = 0.002).

### General composition of the fauna

A total of 40 families, 73 genera and 66 identified species were recovered from the 81 samples (S1 and S2 Tables), with polychaetes and peracarid crustaceans (especially amphipods), being most species-rich (Table 2). Observed richness varied across the sites (Table 2), with the greatest number of identified species being observed off Halifax and the least immediately north of the Orange River (MLA1 South) (Table 2). There were strong, positive and significant (all at p<0.001) relationships between the distribution of richness across sites when measured at the identified species, genus or family level (species-genus R = 0.973, species-family R = 0.944, genus-family R = 0.988), though there was a significant negative relationship between average abundance and diversity per sample (R = 0.93, p<0.05). Greater numbers of identified species per sample were found in the north than south (Table 2) and indeed, the average sample from the former area tended to collect about a third of species present in the communities there, whilst this proportion increased to about half at the southern sites (Table 2). This suggests a greater degree of heterogeneity in the north than in the south.

**Table 2:** Summary of species richness and diversity.

Site	Number species	Number genera	Number families	Mean number species sample^-1^	Max (Min) number species sample^-1^	Amphipods	Isopods	Decapods	Bivalves	Gastropods	Polychaetes	Cnidarians
**Halifax**	36	41	36	10.89	15 (6)	10	1	3	1	1	19	0
**Ebay**	27	28	24	11.33	18 (8)	7	1	2	2	1	13	1
**Bogenfels**	26	27	23	8.11	16 (3)	8	1	1	2	1	12	1
**Chameis**	16	20	19	5.44	12 (2)	5	0	1	0	1	8	1
**Kerbehuk**	28	30	26	9.89	17 (4)	6	1	3	1	1	15	1
**DBMN MA1 North**	25	25	22	13	15 (9)	8	0	2	0	2	12	1
**DBMN MA1 South**	17	18	17	9.56	12 (5)	5	0	3	0	1	8	0
**ML3 North**	22	23	22	10.44	16 (4)	7	0	4	1	1	9	0
**ML3 South**	22	23	22	8.44	12 (5)	5	1	2	1	1	12	0

Observed species richness of the major macro-infauna at each of the sites sampled off southern Namibia and off Namqualand during 2003. The total number of species, genera and families recovered from the nine samples collected from each site also shown, as well as information on the average, maximum and minimum number of species recovered from each sample at each site.

Although polychaetes tended to be the most numerous infauna (Table 3), their dominance and abundance varied so that at, (e.g.) Chameis, bivalves were most common, whilst at ML3 North, peracarid crustaceans were dominant. The most abundant families collected at each site are shown in S3 Table. The polychaete families Spionidae and Magelonidae, and, to a lesser extent, Nephtyidae, Onuphidae and Paraonidae were conspicuous at most sites, whilst Nassariidae (Gastropoda), Tellinidae (Bivalvia), Thalassinidae (Decapoda) and Ampeliscidae (Amphipoda) were also important.

**Table 3:** The abundance of dominant infauna, by lower taxonomic grouping.

Site	Amphipods	Isopods	Decapods	Bivalves	Gastropods	Polychaetes	Cnidarians
**Halifax**	**9.8 (3.2)**	**0.4 (0.4)**	**8.3 (2.5)**	**8.6 (4.2)**	**5.8 (2.5)**	**102.3 (48.6)**	**—-**
**Ebay**	**10.7 (4.5)**	**0.1 (0.1)**	**0.8 (0.3)**	**3.0 (1.2)**	**16.3 (4.1)**	**200.8 (67.0)**	**0.7 (0.3)**
**Bogenfels**	**9.2 (3.9)**	**0.9 (0.3)**	**1.4 (0.8)**	**1.3 (0.5)**	**0.1 (0.1)**	**51.7 (19.7)**	**1.0 (0.5)**
**Chameis**	**2.2 (0.7)**	**0.1 (0.1)**	**0.3 (0.2)**	**359.9 (105.3)**	**5.4 (1.9)**	**5.0 (1.5)**	**1.3 (0.6)**
**Kerbehuk**	**85.4 (31.3)**	**0.2 (0.2)**	**0.9 (0.3)**	**1.4 (0.6)**	**42.0 (16.1)**	**190.9 (59.8)**	**0.3 (0.2)**
**DBMN MA1 North**	**9.0 (1.8)**	**—-**	**17.3 (1.6)**	**0.9 (0.7)**	**0.8 (0.4)**	**45.4 (5.4)**	**0.1 (0.1)**
**DBMN MA1 South**	**19.3 (1.7)**	**—-**	**12.1 (1.6)**	**0.1 (0.1)**	**19.1 (3.3)**	**26.8 (6.7)**	**—-**
**ML3 North**	**26.6 (15.3)**	**—-**	**6.6 (2.2)**	**3.4 (1.3)**	**0.3 (0.3)**	**22.9 (6.3)**	**—-**
**ML3 South**	**4.9 (1.7)**	**0.3 (0.3)**	**1.3 (0.9)**	**5.6 (2.4)**	**1.2 (0.6)**	**59.2 (17.1)**	**—-**

Mean abundance of major macro-infauna found at each of the sites sampled off southwestern Africa during 2003. Data as mean ± standard error, N = 9, in all cases.

### Environment-fauna relations

**Fig 3:**
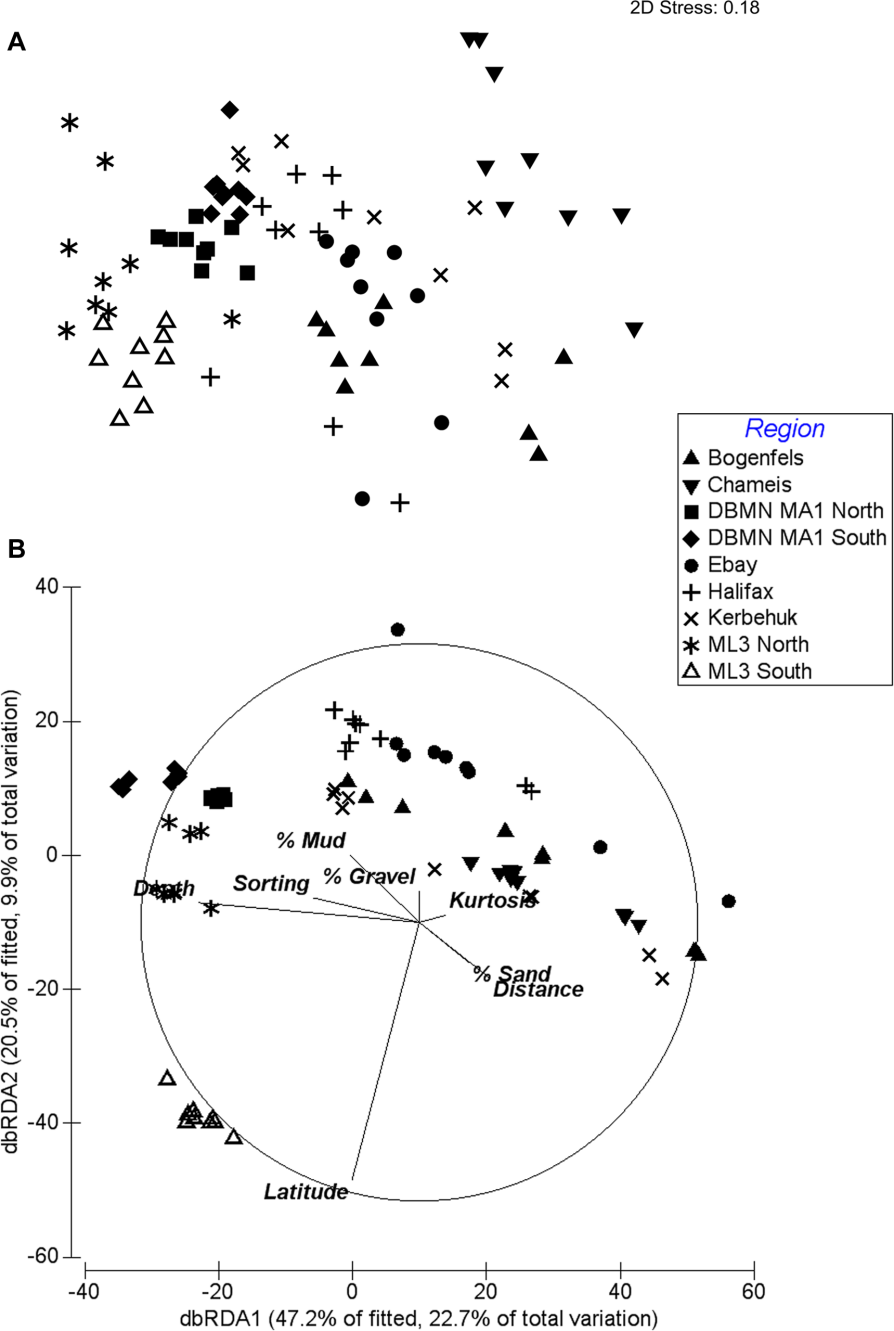
Two-dimensional visualisations of faunal communities. **(A)** MDS plot of the similarity in the species composition of macrofaunal (> 1mm length) samples collected off southern Namibia and off Namaqualand during 2003. (B) Multivariate multiple regression dbRDA performed on the macrofaunal species composition of samples collected off southern Namibia and off Namaqualand during 2003, and environmental predictors: vectors show the direction and strength of the environmental gradients. The location of the samples, by site, is indicated by symbols.

Samples from the shallower mid-shelf sites group to the right of the MDS plot (Fig 3A), whilst those from the deeper sites can be seen to the left. In general, samples from the same site tend to group fairly close together. Patterns were clearer when the data were analysed separately by identified species (stress value = 0.18), than by genus (0.19) or family (0.22), although there was a high level of concordance between the similarity matrices, as determined by the relate statistic (species-genus R = 0.932, species-family R = 0.859, genus-family R = 0.933). The results of the marginal DistLM tests on the identified species-level data indicate that all but one (% gravel) of the predictor variables had a significant impact on the structure of the communities (with water-depth, latitude/site and distance from the Orange River being most important, followed by mud and sand content respectively). And though the overall model included all variables, it could only explain 42.2% of the pattern (Table 4). This is shown graphically in the dbRDA plot (Fig 3B), the first two (of eight) axes of which explain ~67% of fitted variation and ~33% of total variation, and separate samples by water-depth (dbRDA1) and latitude/site (dbRDA2). Essentially similar patterns are shown whether the data are analysed by genus (DistLM adj R^2^ = 0.42: data not shown) or family (DistLM adj R^2^ = 0.40: data not shown). Interestingly, there appears to be less variability between samples within sites, from the deeper than shallower areas.

**Table 4:** Results of the DistLM marginal and sequential tests for the abundance of macro-infauna collected off southwestern Africa during 2003, with sediment texture characteristics, water-depth, latitude/site and distance from the Orange River mouth as predictors.

**Marginal Tests**				
**Variable**		**SS (trace)**	**Pseudo-F**	**p**
**Sorting**		25335	9.5521	0.001
**Skewness**		9236.3	3.2338	0.002
**Kurtosis**		24380	9.1504	0.001
**Distance from Orange River**		28214	10.786	0.001
**% Mud**		23529	8.7953	0.001
**% Sand**		19522	7.1617	0.001
**% Gravel**		3736.4	1.2771	0.221
**Depth**		50484	21.63	0.001
**Latitude**		33303	13.052	0.001
**Sequential Tests**				
**Variable**	**Adj R** ^**2**^	**SS (trace)**	**Pseudo-F**	**p**
**Depth**	0.20501	50484	21.63	0.001
**Latitude**	0.2785	19164	9.0471	0.001
**Distance**	0.35375	19130	10.083	0.001
**% Sand**	0.37623	6912.9	3.7749	0.001
**Kurtosis**	0.39624	6237.4	3.5188	0.003
**% Mud**	0.41306	5427.5	3.1497	0.002
**% Gravel**	0.42168	3570.6	2.103	0.025
**Sorting**	0.42226	1818.5	1.0721	0.375

Sequential tests were conducted using the ‘step-wise’ procedure and adjusted R^2^. Sediment size fractions were arcsine transformed, and all other variables log_10_ transformed, prior to analysis. Residual DF = 79.

**Fig 4:**
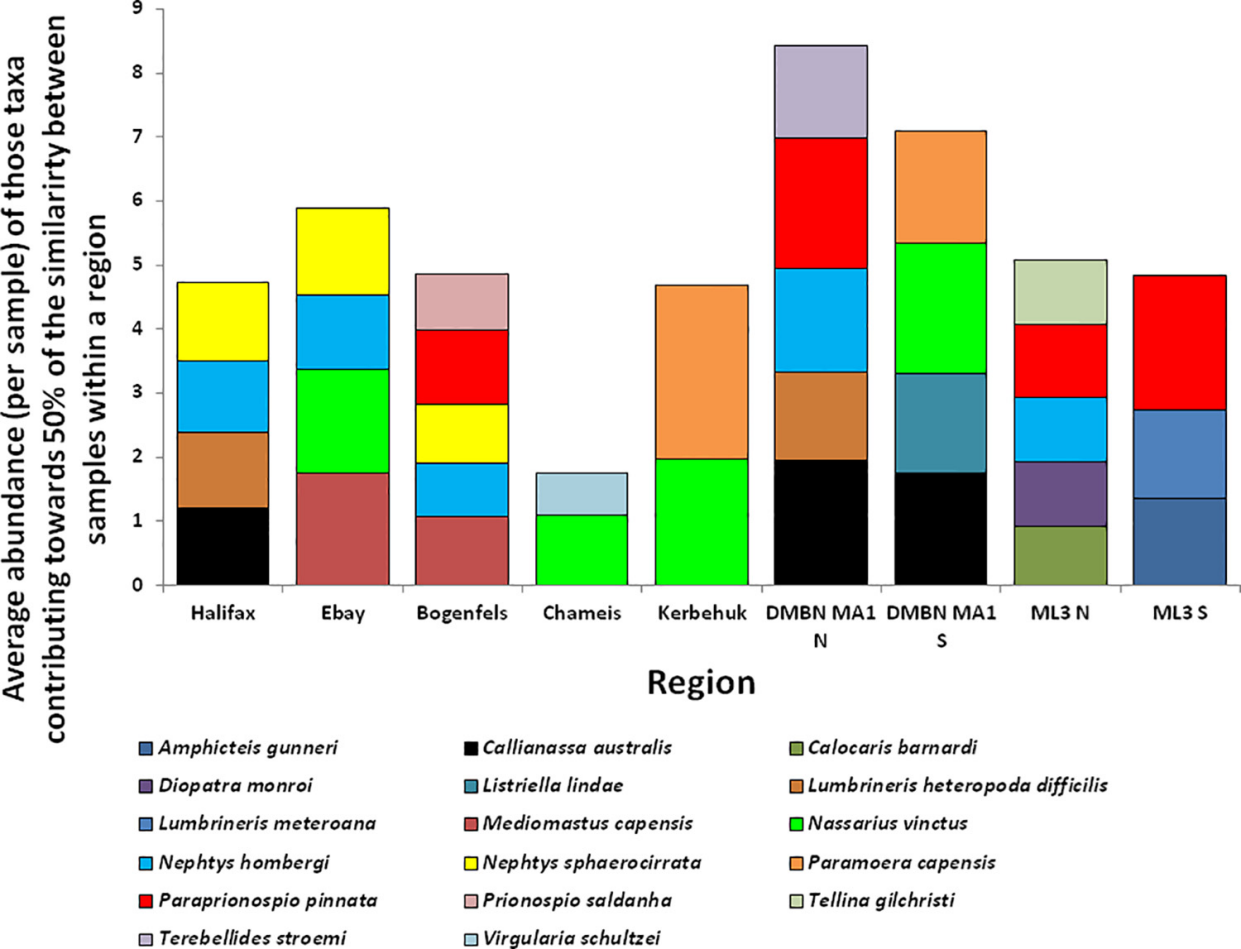
Abundance of those taxa characteristic of each site. Histogram showing the average abundance per site, of those macro-infaunal taxa identified by the SIMPER routine in PRIMER 6 as being responsible for a minimum of 50% of the identity of each of the sites sampled off southern Namibia and off Namaqualand during 2003.

In an attempt to understand the relatively low proportion of the variability in infaunal structure explained by the environmental predictors, we have looked at the results of the SIMPER analysis and the average abundance per sample of those species responsible for 50% of the similarity within sites. These data are shown in Fig 4, from which it is clear that many of the “identifiers” are shared across sites. For example, the polychaete *Nepthys hombergi* was common at five sites, whilst the polychaete *Paraprionospio pinnata* and the gastropod *Nassarius vinctus* were common at four. Indeed, almost 50% of identified species were recovered at, and dominant at, three or more of the sites (S2A Fig). The results of a similar analysis conducted with respect to water-depth and sediment are shown in S4 and S5 Tables, from which it can be seen, especially with respect to water-depth (S4 Table), that although there were clear patterns in species distribution across the shelf, relatively few species were restricted to single habitats. In other words, the fauna of the region appears to be dominated by habitat generalists and differences between sites largely reflect differences in relative abundance.

### How many species?

Estimates of amphipod and polychaete richness at each site, as determined using CurveFit, were positively correlated with observed richness (R = 0.95 p<0.001, R = 0.68 p<0.05 respectively), being highest for both taxa at Halifax and lowest at the sites closer to the Orange River (Table 5). Similar results were observed for estimates of amphipod and polychaete richness at the genus level (R = 0.93 p<0.01, R = 0.83 p<0.01 respectively). There was, however, no relationship between the estimated or observed richness of amphipods or polychaetes and sediment textural diversity at each site (p>0.05). The total number of amphipod and polychaete species estimated for the whole region is 43 and 44 respectively, which is between 150–200% higher than the observed totals (Table 5). The estimates of generic richness are higher still (48 and 71 genera respectively), suggesting that much work needs still to be done to identify species fully. Note the higher numbers of taxa estimated from Halifax than from the region as a whole. This can be attributed to the smaller number of samples collected at Halifax relative to overall, the higher degree of heterogeneity in composition of samples collected at Halifax and the high degree of similarity in the composition between sites.

**Table 5:** How many species?

	Species				Genus			
	Amphipods		Polychaetes		Amphipods		Polychaetes	
Region	Estimated	Observed	Estimated	Observed	Estimated	Observed	Estimated	Observed
**Halifax**	68	14	278	22	146	11	404	21
**Ebay**	33	8	31	14	11	7	23	12
**Bogenfels**	42	10	73	13	77	9	10	9
**Chameis**	8	7	14	10	7	6	12	9
**Kerbe Huk**	9	6	17	17	9	6	18	15
**DBMN MA1 North**	35	8	59	14	19	6	42	12
**DBMN MA1 South**	6	5	80	8	6	5	24	7
**ML3 North**	9	7	9	10	8	6	9	9
**ML3 South**	6	6	16	13	5	4	15	12
**ALL**	**43**	**22**	**44**	**31**	**48**	**21**	**71**	**32**

Observed and estimated species and generic richness of amphipods and polychaetes at each of the sites sampled off southern Namibia and off Namaqualand during 2003. Regional data also shown. Estimated richness determined using Curve Fit estimator and Morgan-Mecer-Flodin model (see methods).

### Geographic affinities of fauna

All of the identified species recovered here have records in one or more of the species distribution databases analysed, and 89% have previously been recorded in Namibia (S1 Table). The majority are widely distributed in the Atlantic (30%) or along the coasts of Benguela nations (Angola, Namibia and South Africa—26%), and some 15% can be regarded as southern African and extend around South Africa to Mozambique and the wider Indian Ocean. Seventeen percent of species occur widely in both the Atlantic and Indian oceans. Merely ~10% enjoys a global distribution and only two species are restricted to Namibia (~3%): the brachiopod *Discinisca tenuis* and the polychaete *Pterampharete luederitzi*. These species were first described from outside the study area, and were recovered here from one and five samples (respectively), at a single site each (Halifax and Kerbehuk, respectively).

## Discussion

The mud content of the sediments tended to decrease with increasing distance from the Orange River (Fig 2B), especially northwards from the Orange River, and sediments were most gravelly in the north (Table 1). This tallies with the results of previous regional surveys [22, 24]. The remarkably poor correlation (29%) between sediment texture vs distance from the Orange River mouth, latitude and water-depth may be explained by the lack of an inner-shelf mudbelt between Chameis Bay and Lüderitz. Had the study been undertaken only south of Chameis Bay, where there is a major inner-shelf mudbelt, a better correlation may well have been observed.

The fauna was dominated by polychaetes and crustaceans (especially peracarids), although bivalves were abundant at one of the sites. These taxa typically characterise the infauna of soft sediments everywhere [45], and may be supplemented with echinoderms, which were largely absent here. In their study further north along the Namibian coast and off southern Angola, Zettler et al. [8] noted nearshore communities to be dominated by brachiopods (the endemic *Discinisca tenuis* collected here), whilst those within the OMZ further offshore were dominated by bivalves (*Nuculana bicuspidata*). This is unusual for OMZs, which are typically characterised by small-bodied polychaetes [7], mostly from the families Cossuridae [9], Spionidae [46], Cirratulidae [47], Paraonidae, Amphinomidae, Maldanidae [7], and Magelonidae [9, 48]. Other taxa that may be common include ampeliscid amphipods [7] and nassariid gastropods [8–9]. That many of the dominant taxa observed here are not only found in these same families, but may in fact be the same species as those from OMZs elsewhere in the world (e.g. *Paraprionospio pinnata*), indicates that the environment is certainly oxygen stressed, especially in deeper waters. However, the presence of species such as the relatively long-lived *Callianassa australis*, which builds deep burrows in the sediment [49–50], suggests it is not routinely microxic across the region.

The high level of concordance between the ecological patterns generated by the analyses (similarity matrices, outputs of the DistLMs) conducted on, and the positive relationships between, the different levels of taxonomic resolution has been noted previously [51–52]. And it is often cited as a reason to study communities at lower taxonomic levels of classification [53]. In this case, the analyses at the different levels of taxonomic resolution included different amounts of the full dataset, with those at the family-level including more than those at the genus-level, which in turn included more than those at the species-level.

The results indicate that of the measured environmental variables, water-depth, latitude and distance from the Orange River mouth were most important in explaining variation in the structure of the infaunal communities, with the sediment texture playing a secondary role. There is a wide body of literature indicating that both water depth and latitude influence communities through their effect on the regional species pool [17] and, in the case of water-depth, on the texture of the sediments [22, 24, 27]. However, we suggest (below) that the latitudinal impact on community composition, *per se*, is negligible, which means that it acts here through its influence on water-depth and sediment texture: samples from deeper water were collected in the south, off Namaqualand, and these samples tended to be muddier than the shallower samples collected in the north. Although most authors agree that sediment texture generally plays a key role in influencing community structure (e.g. [54]), concordance between the distribution of sediment types and biological communities is often not clear (e.g. [55–57]). Such was apparent here, where the adjusted R^2^ of the DistLM of the species-level data was <0.4. This implies that the variability in the biological communities must largely be due to spatio-temporal differences in unmeasured variables such as (e.g.) dissolved oxygen [7] and recruitment [58], as well as food availability and disturbance [59], and metal concentrations [60], all of which are known to impact communities, individually and in synergy (see reviews by [61–62]). Of course, variations in biological interactions (directly and/or indirectly) should not be ignored [62]. For example, biogenic structures may increase habitat complexity and environmental heterogeneity, promoting diversity and co-existence through non-equilibrium mechanisms (as e.g. *Callaniassa* burrows [49–50]). Regardless, the high variability observed in the communities suggests that the study area as a whole falls outside an OMZ, where low levels of biological heterogeneity are the norm [7].

Despite a strong latitudinal pattern to the structure of the biological communities observed here, most of the dominant taxa were widely distributed across the sites and differed, for the most part, simply in relative abundance. This suggests that all are drawn from the same regional species pool. That many taxa were distributed widely across the different sediment types, and across the water-depth gradient too, further suggests that most can probably be regarded as habitat generalists. Tropical communities are often considered to have species that are “tightly packed”, with pronounced partitioning of spatial and trophic resources, and such communities are characterised by specialists ([3]: Table 1). By contrast, taxa from higher latitudes often have broader niches than those at the equator, and communities are characterised by generalists ([3]: Table 1). Pelagic communities in upwelling areas are also characterised by generalists [2], which can probably be attributed to environmental variability. And it would seem that similar observations hold in the benthos off Namibia and off Namaqualand outside of OMZs too: presumably for the same reasons.

As stressed by [17], the local community represents a subset of the regional species pool, and in this case the regional species pool is substantial, encompassing the North and South Atlantic Ocean and the Mediterranean Sea, as well as the Western Indian Ocean. Unless the taxonomy is at fault, of course, or if there is significant taxonomic crypsis [63]! Whilst the latter cannot be ruled out, and is certainly deserving of future attention, as it stands the number of endemic species is negligible. Whilst this is in agreement with the observations of [64] on the distribution of regional range-restricted taxa, it is in contrast to the contentions of [65], and to the examples cited by Levin [7]. But that could be explained by the fact that the areas sampled fall outside the main OMZ off Namibia. Of course, it can also be explained by the fact that a number of taxa were not identified to species level, and some of these may yet turn out to be endemic [7].

Despite the size of the potential regional source-pool, the diversity of species in both local samples and communities is remarkably low. Caution should be exercised in interpreting this given unidentified taxa, but it accords with both the generalist nature of community members, the variability of upwelling communities and it confirms what we understand about diversity gradients around the region (e.g. [19, 21]). The negative relationship between average faunal diversity per sample per site and total abundance conforms to the results of many previous studies [45], although the absence of a relationship between either diversity per sample per site, or estimated richness per site, and sediment heterogeneity (as measured by sediment diversity) is unusual. This can probably be attributed to the generalist nature of the fauna and the unmeasured environment.

The results of this study suggest that the fauna of the soft-sediment environment off southern Namibia and off Namqualand is characterised by relatively low diversity and by species with a generalist habit and wide distribution. Further work on measuring a wider suite of environmental variables is desirable, and studies into the detailed taxonomic identity of all material collected is encouraged.

## Supporting Information

S1 Fig Histograms showing the proportion of samples collected at each site off southwestern Africa during 2003, described by Folk’s (1954) sediment textural groups. (TIF)

S2 Fig Distribution of species across sites and sediments. Number (percent of total) of identified species recovered in one or more of the nine regions (a) or sediment textural groups (b) sampled off southern Namibia and off Namaqualand during 2003.
(PPTX)

S1 Table Species List. List of the identified species recovered from the samples collected off southern Namibia and off Namaqualand during 2003, with an indication of their presence (1) or absence (0) in on-line distributional databases (WoRms Wold Register of Marine Species; EoL Encyclopaedia of Life; OBIS Ocean Biogeographic Information System; ERMS European Register of Marine Species). Information from other sources also indicated, where appropriate. A summary of the known distribution of each species is also indicated.
(DOCX)

S2 Table Additional genera. List of additional genera, not included in Table 1 owing to our inability to ascribe specific epithets to names, recovered from the samples collected off southern Namibia and off Namaqualand during 2003, with an indication of their presence (X) or absence (—-) in the different sampled regions.
(DOCX)

S3 Table The abundance of common infaunal families. Mean abundance and standard error (per sample) of the top ten most commonly represented families at each of the sites sampled off southwestern Africa during 2003.
(DOCX)

S4 Table Abundance of those taxa characteristic of each depth class. Average (root-root) abundance per sample of those macro-infaunal taxa identified by the SIMPER routine in PRIMER 6 as being responsible for 90% of the identity of each of the water-depth zones (0–20 m, 21–30 m, 31–50 m, 51–100 m, 101–150 m). The weighted mean water-depth (m) occupied by each of the identified species is also shown.
(DOCX)

S5 Table Abundance of those taxa characteristic of each sediment type. Average (root-root) abundance per sample of those macro-infaunal taxa identified by the SIMPER routine in PRIMER 6 as being responsible for 90% of the identity of each of the sediment textural groups identified by Folk (1954) sampled off southwestern Africa during 2003. The weighted mean sediment particle size (μm) occupied by each of the identified species is also shown.
(DOCX)

S6 Table Abundance of all identified species from all grab samples collected at all sites, together with pertinent bathymetric, geographic and sediment data. (XLSX)
