# Supplementary figures and images for: Infaunal Benthic Communities from the Inner Shelf off Southwestern Africa Are Characterised by Generalist Species
Source: PLoS One. 2015 Nov 30;10(11):e0143637. doi: 10.1371/journal.pone.0143637 (PMC4664413; doi:10.1371/journal.pone.0143637)

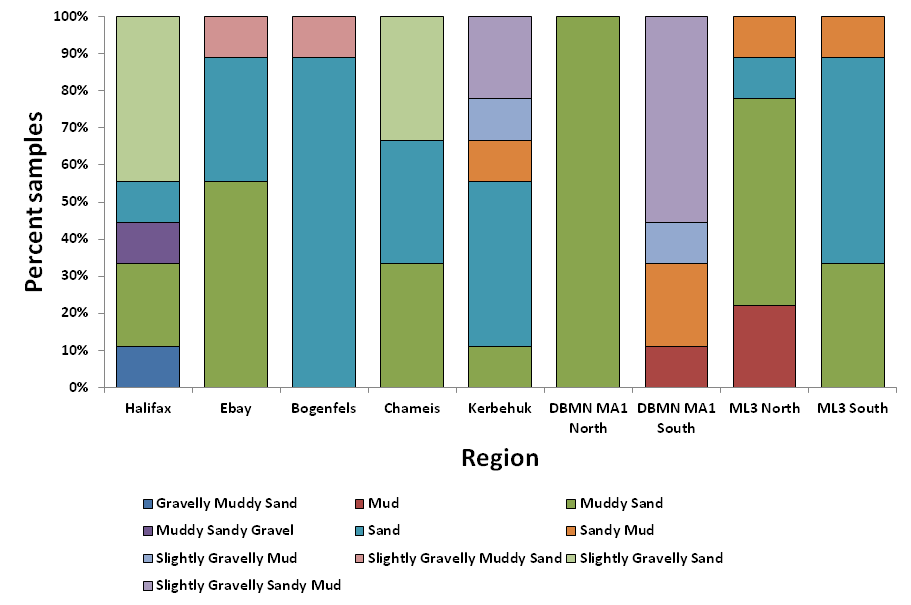

Supplement: S1 Fig — (TIF) [file pone.0143637.s001.tif]
