# Supplementary material for: Infaunal Benthic Communities from the Inner Shelf off Southwestern Africa Are Characterised by Generalist Species
Source: PLoS One. 2015 Nov 30;10(11):e0143637. doi: 10.1371/journal.pone.0143637 (PMC4664413; doi:10.1371/journal.pone.0143637)
Supplement: S2 Fig — Number (percent of total) of identified species recovered in one or more of the nine regions (a) or sediment textural groups (b) sampled off southern Namibia and off Namaqualand during 2003. (PPTX) [file pone.0143637.s002.pptx]

## Slide 1
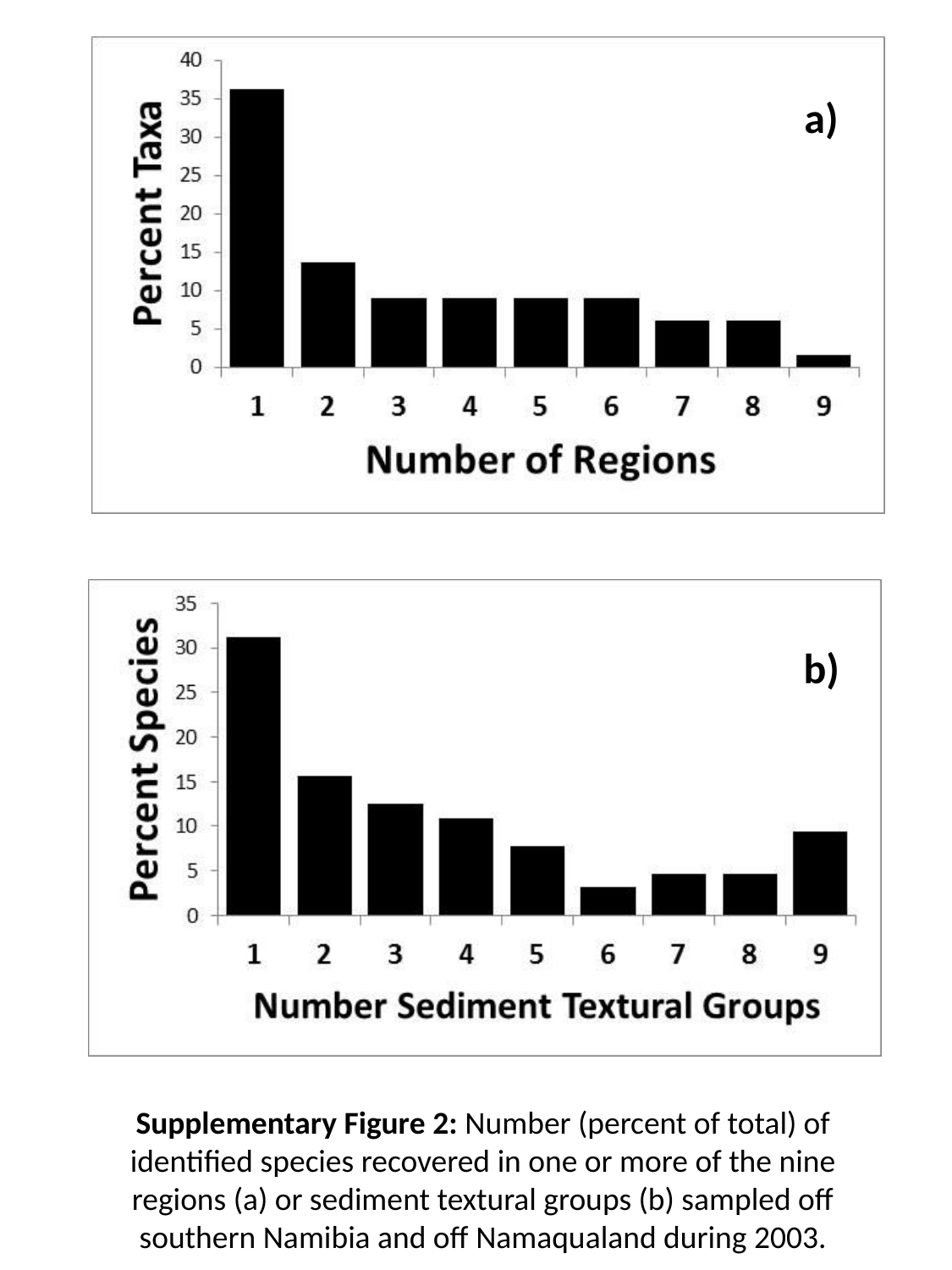

a)
b)
Supplementary Figure 2: Number (percent of total) of identified species recovered in one or more of the nine regions (a) or sediment textural groups (b) sampled off southern Namibia and off Namaqualand during 2003.
