# Supplementary material for: Infaunal Benthic Communities from the Inner Shelf off Southwestern Africa Are Characterised by Generalist Species
Source: PLoS One. 2015 Nov 30;10(11):e0143637. doi: 10.1371/journal.pone.0143637 (PMC4664413; doi:10.1371/journal.pone.0143637)
Supplement: S1 Table — List of the identified species recovered from the samples collected off southern Namibia and off Namaqualand during 2003, with an indication of their presence (1) or absence (0) in on-line distributional databases (WoRms Wold Register of Marine Species; EoL Encyclopaedia of Life; OBIS Ocean Biogeographic Information System; ERMS European Register of Marine Species). Information from other sources also indicated, where appropriate. A summary of the known distribution of each species is also indicated. (DOCX) [file pone.0143637.s003.docx]

| **Species** | **WoRMS** | **EoL** | **OBIS** | **ERMS** | **OTHER** | **Distribution** |
| --- | --- | --- | --- | --- | --- | --- |
| ***Ampelisca anisuropa*** | 1 | 1 | 1 | 0 | 0 | Benguela |
| ***Ampelisca anomala*** | 1 | 1 | 1 | 1 | 0 | Atlantic |
| ***Ampelisca brevicornis*** | 1 | 1 | 1 | 1 | 0 | Atlantic |
| ***Ampelisca palmata*** | 1 | 1 | 1 | 0 | 0 | Regional |
| ***Ampelisca spinimana*** | 1 | 1 | 1 | 1 | 0 | Atlantic |
| ***Amphicteis gunneri*** | 1 | 0 | 1 | 1 | 0 | Global |
| ***Aora kergueleni*** | 1 | 1 | 1 | 0 | 0 | Global |
| ***Aricidea longobranchiata*** | 0 | 0 | 1 | 1 | 0 | Atlantic |
| ***Callianassa australis*** | 1 | 1 | 1 | 0 | 1 | Benguela |
| ***Calocaris barnardi*** | 1 | 1 | 1 | 0 | 0 | Atlantic |
| ***Centranthura caeca*** | 0 | 0 | 1 | 0 | 1 | Benguela |
| ***Corophium triaenonyx*** | 1 | 1 | 1 | 0 | 0 | Regional |
| ***Cossura coasta*** | 1 | 1 | 1 | 1 | 0 | Atlantic |
| ***Diopatra monroi*** | 0 | 0 | 1 | 1 | 0 | Atlantic & Indian |
| ***Discinisca tenuis*** | 0 | 0 | 1 | 0 | 1 | Namibia |
| ***Drilonereis monroi*** | 0 | 1 | 1 | 0 | 0 | Atlantic |
| ***Glycera alba*** | 1 | 0 | 1 | 1 | 0 | Atlantic & Indian |
| ***Glycera benguellana*** | 0 | 1 | 1 | 0 | 0 | Regional |
| ***Glycera convoluta*** | 1 | 0 | 1 | 1 | 0 | Atlantic & Indian |
| ***Goneplax angulata*** | 1 | 1 | 1 | 1 | 0 | Atlantic |
| ***Haploscoloplos kerguelensis*** | 1 | 1 | 1 | 1 | 0 | Global |
| ***Heterophoxus opus*** | 0 | 1 | 1 | 0 | 0 | Benguela |
| ***Hippomedon onconotus*** | 1 | 1 | 1 | 0 | 0 | Regional |
| ***Indischnopus capensis*** | 0 | 0 | 1 | 0 | 1 | Benguela |
| ***Jasmineira elegans*** | 1 | 1 | 1 | 1 | 0 | Atlantic |
| ***Laetmatophilus purus*** | 1 | 1 | 1 | 1 | 0 | Atlantic & Indian |
| ***Leucothoe richiardi*** | 1 | 1 | 1 | 1 | 0 | Atlantic & Indian |
| ***Listriella lindae*** | 0 | 0 | 1 | 0 | 0 | Benguela |
| ***Lumbrineris heteropoda difficilis*** | 0 | 0 | 1 | 0 | 0 | Benguela |
| ***Lumbrineris meteroana*** | 0 | 0 | 0 | 0 | 1 | Atlantic & Indian |
| ***Lumbrineris tetraura*** | 1 | 1 | 1 | 1 | 0 | Atlantic |
| ***Lysianassa variegata*** | 1 | 1 | 0 | 0 | 0 | Regional |
| ***Mediomastus capensis*** | 1 | 1 | 1 | 1 | 0 | Atlantic |
| ***Megaluropus namaquaeensis*** | 1 | 1 | 1 | 0 | 0 | Benguela |
| ***Nassarius vinctus*** | 1 | 1 | 1 | 0 | 0 | Benguela |
| ***Nautilocorystes ocellata*** | 1 | 1 | 1 | 0 | 0 | Atlantic |
| ***Nephtys hombergi*** | 1 | 0 | 1 | 1 | 0 | Atlantic |
| ***Nephtys sphaerocirrata*** | 1 | 1 | 1 | 1 | 0 | Atlantic & Indian |
| ***Nerinides gilchristi*** | 0 | 1 | 1 | 1 | 0 | Atlantic |
| ***Onuphis holobranchiata*** | 0 | 1 | 1 | 0 | 0 | Global |
| ***Orbinia angrapequensis*** | 0 | 0 | 1 | 0 | 0 | Benguela |
| ***Owenia fusiformis*** | 1 | 0 | 1 | 1 | 0 | Global |
| ***Paracabira capensis*** | 0 | 1 | 1 | 0 | 0 | Benguela |
| ***Paramoera capensis*** | 1 | 1 | 1 | 0 | 0 | Atlantic |
| ***Paraprionospio pinnata*** | 1 | 1 | 1 | 1 | 0 | Atlantic & Indian |
| ***Pectinaria capensis*** | 1 | 1 | 1 | 0 | 0 | Regional |
| ***Perioculodes longimanus*** | 1 | 1 | 1 | 1 | 0 | Atlantic & Indian |
| ***Perioculodes pallidus*** | 1 | 1 | 1 | 0 | 0 | Benguela |
| ***Phaxas decipiens*** | 1 | 0 | 1 | 0 | 0 | Benguela |
| ***Pherusa swakopiana*** | 0 | 0 | 1 | 0 | 0 | Benguela |
| ***Photis longidactylus*** | 0 | 0 | 0 | 0 | 1 | Regional |
| ***Photis longimanus*** | 1 | 1 | 0 | 0 | 1 | Regional |
| ***Prionospio saldanha*** | 1 | 1 | 1 | 0 | 0 | Atlantic |
| ***Prionospio sexoculata*** | 1 | 1 | 1 | 1 | 0 | Atlantic |
| ***Pterygosquilla armata capensis*** | 1 | 1 | 1 | 0 | 0 | Benguela |
| ***Sabellides luderitzi*** | 0 | 0 | 0 | 0 | 1 | Namibia |
| ***Sigambra parva*** | 1 | 1 | 1 | 1 | 0 | Atlantic & Indian |
| ***Synidotea hirtipes*** | 1 | 1 | 1 | 0 | 0 | Regional |
| ***Tellina gilchristi*** | 1 | 1 | 1 | 0 | 0 | Regional |
| ***Telothelepus capensis*** | 1 | 1 | 1 | 0 | 0 | Atlantic |
| ***Terebellides stroemi*** | 1 | 1 | 1 | 1 | 0 | Global |
| ***Urothoe grimaldi*** | 1 | 1 | 1 | 1 | 0 | Atlantic & Indian |
| ***Virgularia schultzei*** | 1 | 1 | 1 | 0 | 0 | Benguela |
| ***Volutocorbis lutosa*** | 1 | 1 | 1 | 0 | 0 | Atlantic |
| ***Westwoodilla manta*** | 0 | 1 | 1 | 0 | 0 | Benguela |

**Supplementary Table 1:** List of the identified species recovered from the samples collected off southern Namibia and off Namaqualand during 2003, with an indication of their presence (1) or absence (0) in on-line distributional databases (WoRms Wold Register of Marine Species; EoL Encyclopaedia of Life; OBIS Ocean Biogeographic Information System; ERMS European Register of Marine Species). Information from other sources also indicated, where appropriate. A summary of the known distribution of each species is also indicated.
