# Supplementary material for: Infaunal Benthic Communities from the Inner Shelf off Southwestern Africa Are Characterised by Generalist Species
Source: PLoS One. 2015 Nov 30;10(11):e0143637. doi: 10.1371/journal.pone.0143637 (PMC4664413; doi:10.1371/journal.pone.0143637)
Supplement: S2 Table — List of additional genera, not included in Table 1 owing to our inability to ascribe specific epithets to names, recovered from the samples collected off southern Namibia and off Namaqualand during 2003, with an indication of their presence (X) or absence (—-) in the different sampled regions. (DOCX) [file pone.0143637.s004.docx]

| **Site** | **Halifax** | **Ebay** | **Bogenfels** | **Chameis** | **Kerbehuk** | **DBMN MA1 North** | **DBMN MA1 South** | **ML3 North** | **ML3 South** |
| --- | --- | --- | --- | --- | --- | --- | --- | --- | --- |
| ***Cirolana*** | --- | --- | --- | X | --- | --- | --- | --- | --- |
| ***Cirriformia*** | --- | --- | --- | --- | --- | --- | --- | --- | X |
| ***Eulalia*** | X | --- | --- | --- | --- | --- | --- | --- | --- |
| ***Eurydice*** | --- | --- | X | --- | --- | --- | --- | --- | --- |
| ***Gammaropsis*** | X | --- | --- | --- | --- | --- | --- | --- | --- |
| ***Glycinde*** | X | --- | --- | --- | --- | --- | --- | --- | --- |
| ***Goniadia*** | --- | --- | --- | --- | --- | --- | --- | --- | X |
| ***Harmothoe*** | --- | --- | --- | --- | X | --- | --- | --- | --- |
| ***Lepidepecreum*** | --- | --- | --- | --- | --- | --- | --- | --- | X |
| ***Melita*** | X | --- | --- | --- | --- | --- | --- | --- | --- |
| ***Syllis*** | X | --- | --- | --- | --- | --- | --- | --- | --- |
| ***Bathyporeia*** | X | X | --- | --- | --- | --- | --- | --- | --- |
| ***Atylus*** | X | --- | X | X | --- | --- | --- | --- | --- |
| ***Nereis*** | --- | --- | --- | --- | --- | X | --- | X | X |
| ***Pasiphae*** | --- | --- | --- | --- | --- | X | X | X | X |
| ***Dosinia*** | X | X | X | --- | X | --- | --- | X | X |
| ***Magelona*** | X | X | X | X | X | X | --- | --- | --- |
| ***Macoma*** | X | X | X | X | X | X | X | --- | --- |
