# Supplementary material for: Infaunal Benthic Communities from the Inner Shelf off Southwestern Africa Are Characterised by Generalist Species
Source: PLoS One. 2015 Nov 30;10(11):e0143637. doi: 10.1371/journal.pone.0143637 (PMC4664413; doi:10.1371/journal.pone.0143637)
Supplement: S3 Table — Mean abundance and standard error (per sample) of the top ten most commonly represented families at each of the sites sampled off southwestern Africa during 2003. (DOCX) [file pone.0143637.s005.docx]

| **Taxon** | **Family** | **Halifax** | **Ebay** | **Bogenfels** | **Chameis** | **Kerbehuk** | **DBMN MA1 North** | **DBMN MA1 South** | **ML3 North** | **ML3 South** |
| --- | --- | --- | --- | --- | --- | --- | --- | --- | --- | --- |
| **Amphipoda** | **Ampeliscidae** | -- | 3.4 (1.2) | 3.0 (1.2) | -- | -- | 2.2 (0.9) | 2.8 (0.8) | 22.6 (13.4) | 2.8 (1.0) |
|  | **Bathyporeiidae** | -- | 1.8 (1.8) | -- | -- | -- | -- | -- | -- | -- |
|  | **Corophiidae** | -- | -- | -- | -- | -- | -- | -- | 2.4 (1.8) | -- |
|  | **Eusiridae** | -- | -- | -- | 0.9 (0.5) | 82.3 (31.4) | -- | 10 (1.6) | -- | -- |
|  | **Haustoriidae** | -- | 4.2 (2.9) | 4.1 (2.1) | 0.6 (0.3) | 2.1 (1.5) | -- | -- | -- | -- |
|  | **Liljeborgiidae** | -- | -- | -- | -- | -- | 4.2 (1.0) | 6.1 (0.9) | -- | -- |
|  | **Oedicerotidae** | -- | -- | 1.3 (0.9) | -- | -- | 2.0 (0.7) | -- | -- | -- |
| **Bivalvia** | **Tellinidae** | 5.7 (2.7) | 1.6 (0.8) | 1.1 (0.4) | 359.9 (105.3) | -- | -- | -- | 2.9 (1.1) | 4.0 (2.4) |
|  | **Veneridae** | -- | -- | -- | -- | -- | -- | -- | -- | 1.6 (0.7) |
| **Cnidaria** | **Virgulariidae** | -- | -- | -- | 1.3 (0.6) | -- | -- | -- | -- | -- |
| **Decapoda** | **Axiidae** | -- | -- | -- | -- | -- | -- | -- | 2.6 (0.8) | -- |
|  | **Squillidae** | -- | -- | -- | -- | -- | 1.7 (0.3) | 1.1 (0.3) | -- | -- |
|  | **Thalassinidea** | 7.9 (2.5) | -- | 1.4 (0.7) | -- | -- | 14.7 (1.4) | 10.4 (1.5) | -- | -- |
|  | **Thiidae** | -- | -- | -- | 0.3 (0.2) | -- | -- | -- | -- | -- |
| **Gastropoda** | **Nassariidae** | 5.8 (2.5) | 16.3 (4.1) | -- | 5.4 (1.9) | 42.0 (16.0) | -- | 19.1 (3.3) | -- | -- |
| **Polychaeta** | **Ampharetidae** | -- | -- | -- | -- | 3.1 (1.9) | -- | -- | -- | 5.4 (1.5) |
|  | **Capitellidae** | -- | 14.7 (4.5) | 5.0 (1.7) | -- | 3.4 (1.6) | -- | -- | -- | -- |
|  | **Cirratulidae** | -- | -- | 3.7 (1.6) | 0.7 (0.4) | -- | -- | -- | -- | -- |
|  | **Cossuridae** | -- | -- | -- | -- | 1.8 (0.7) | -- | -- | -- | -- |
|  | **Glyceridae** | -- | -- | -- | 0.8 (0.3) | -- | -- | -- | -- | -- |
|  | **Lumbrineridae** | 5.7 (2.2) | -- | -- | -- | -- | 7.2 (1.0) | -- | 3.2 (1.2) | 6.3 (2.3) |
|  | **Magelonidae** | -- | 159.4 (62.4) | 20.1 (11.8) | -- | 22.8 (21.7) | -- | -- | -- | -- |
|  | **Maldanidae** | 6.6 (6.4) | -- | -- | -- | -- | -- | -- | 2.2 (1.1) | 2.7 (1.1) |
|  | **Nephtyidae** | 8.7 (2.0) | 10.0 (2.5) | 4.8 (1.4) | -- | 7.6 (1.8) | 7.3 (1.1) | 2.6 (0.8) | 2.6 (0.7) | 2.8 (0.5) |
|  | **Onuphidae** | 22.8 (21.9) | -- | -- | -- | -- | 3.1 (1.3) | 6.7 (1.6) | 2.8 (1.1) | -- |
|  | **Orbiniidae** | -- | -- | -- | -- | -- | -- | -- | -- | 1.8 (0.5) |
|  | **Oweniidae** | 16.1 (16.1) | -- | -- | -- | -- | -- | -- | -- | -- |
|  | **Paraonidae** | 8.7 (4.8) | 7.9 (3.0) | -- | 0.9 (0.6) | 1.2 (0.8) | -- | -- | -- | 2.3 (0.7) |
|  | **Pilargidae** | -- | -- | -- | -- | -- | -- | 1.6 (0.7) | -- | -- |
|  | **Spionidae** | 14.2 (9.0) | 6.6 (1.0) | 17.3 (6.1) | 1.1 (0.5) | 146.7 (52.2) | 19.8 (4.4) | 15.0 (5.5) | 4.1 (1.4) | 33.6 (15.1) |
|  | **Terebellidae** | -- | -- | -- | -- | -- | 4.9 (0.1) | -- | 2.2 (1.0) | -- |
