# Supplementary material for: Infaunal Benthic Communities from the Inner Shelf off Southwestern Africa Are Characterised by Generalist Species
Source: PLoS One. 2015 Nov 30;10(11):e0143637. doi: 10.1371/journal.pone.0143637 (PMC4664413; doi:10.1371/journal.pone.0143637)
Supplement: S4 Table — Average (root-root) abundance per sample of those macro-infaunal taxa identified by the SIMPER routine in PRIMER 6 as being responsible for 90% of the identity of each of the water-depth zones (0–20 m, 21–30 m, 31–50 m, 51–100 m, 101–150 m). The weighted mean water-depth (m) occupied by each of the identified species is also shown. (DOCX) [file pone.0143637.s006.docx]

| **Depth Class (m)** | **20** | **30** | **50** | **100** | **150** | **Weighted Mean Depth (m)** |
| --- | --- | --- | --- | --- | --- | --- |
| ***Ampelisca brevicornis*** | 1.01 | 0 | 0 | 0 | 0 | 20 |
| ***Urothoe grimaldi*** | 1.23 | 0.7 | 0 | 0 | 0 | 24 |
| ***Virgularia schultzei*** | 1.12 | 0.73 | 0.44 | 0 | 0 | 29 |
| ***Prionospio saldanha*** | 1.58 | 0.45 | 0.67 | 0 | 0 | 29 |
| ***Ampelisca spinimana*** | 0 | 0 | 0.46 | 0 | 0 | 50 |
| ***Aricidea longobranchiata*** | 0 | 0 | 0.7 | 0 | 0 | 50 |
| ***Glycera convoluta*** | 0 | 0 | 0.46 | 0 | 0 | 50 |
| ***Mediomastus capensis*** | 0 | 0.47 | 1.22 | 0.59 | 0 | 59 |
| ***Paramoera capensis*** | 0 | 0.99 | 0.57 | 1.04 | 0 | 62 |
| ***Nephtys sphaerocirrata*** | 0 | 0 | 1.08 | 0.86 | 0 | 72 |
| ***Tellina gilchristi*** | 0.83 | 0 | 0 | 0 | 0.58 | 73 |
| ***Nassarius vinctus*** | 0 | 0 | 1.67 | 1.63 | 0 | 75 |
| ***Nephtys hombergi*** | 0 | 0.48 | 0.72 | 1.24 | 1.21 | 98 |
| ***Sigambra parva*** | 0 | 0 | 0 | 0.72 | 0 | 100 |
| ***Paraprionospio pinnata*** | 0 | 0 | 0.68 | 1.86 | 1.69 | 112 |
| ***Listriella lindae*** | 0 | 0 | 0 | 0.95 | 0.62 | 120 |
| ***Lumbrineris heteropoda difficilis*** | 0 | 0 | 0 | 0.89 | 0.6 | 120 |
| ***Callianassa australis*** | 0 | 0 | 0 | 1.32 | 1 | 122 |
| ***Diopatra monroi*** | 0 | 0 | 0 | 0.85 | 0.86 | 125 |
| ***Ampelisca anomala*** | 0 | 0 | 0 | 0 | 0.83 | 150 |
| ***Amphicteis gunneri*** | 0 | 0 | 0 | 0 | 0.58 | 150 |
| ***Lumbrineris meteroana*** | 0 | 0 | 0 | 0 | 1.06 | 150 |
| ***Pterygosquilla armata capensis*** | 0 | 0 | 0 | 0 | 0.55 | 150 |
| ***Terebellides stroemi*** | 0 | 0 | 0 | 0 | 0.79 | 150 |
