# Supplementary material for: Infaunal Benthic Communities from the Inner Shelf off Southwestern Africa Are Characterised by Generalist Species
Source: PLoS One. 2015 Nov 30;10(11):e0143637. doi: 10.1371/journal.pone.0143637 (PMC4664413; doi:10.1371/journal.pone.0143637)
Supplement: S5 Table — Average (root-root) abundance per sample of those macro-infaunal taxa identified by the SIMPER routine in PRIMER 6 as being responsible for 90% of the identity of each of the sediment textural groups identified by Folk (1954) sampled off southwestern Africa during 2003. The weighted mean sediment particle size (μm) occupied by each of the identified species is also shown. (DOCX) [file pone.0143637.s007.docx]

| **Mean Grain Size (μm)** | **38** | **61** | **62** | **156** | **201** | **223** | **297** | **479** | **Weighted Mean Grain Size (μm)** |
| --- | --- | --- | --- | --- | --- | --- | --- | --- | --- |
| **Sediment Texture** | **Mud** | **Sandy Mud** | **Slightly Gravelly Mud** | **Slightly Gravelly Muddy Sand** | **Muddy Sand** | **Slightly Gravelly Sandy Mud** | **Sand** | **Slightly Gravelly Sand** |  |
| ***Calocaris barnardi*** | 1.41 | 0 | 0 | 0 | 0 | 0 | 0 | 0 | 38 |
| ***Pterygosquilla armata capensis*** | 0 | 0 | 1 | 0 | 0.47 | 0 | 0 | 0 | 106 |
| ***Paraprionospio pinnata*** | 1.7 | 2.61 | 3.05 | 2.07 | 1.21 | 1.97 | 0.86 | 0 | 124 |
| ***Diopatra monroi*** | 0 | 0.96 | 1.41 | 0 | 0.59 | 1.31 | 0 | 0 | 130 |
| ***Paramoera capensis*** | 0 | 1.16 | 2.43 | 0 | 0.36 | 1.82 | 0.48 | 0 | 134 |
| ***Sigambra parva*** | 0.86 | 0.88 | 0 | 0 | 0 | 0 | 0 | 0.55 | 152 |
| ***Nassarius vinctus*** | 1.16 | 1.58 | 2.61 | 0 | 0.62 | 2.23 | 0.57 | 1.72 | 183 |
| ***Nephtys hombergi*** | 0 | 1.13 | 1.37 | 1.09 | 1.17 | 1.19 | 0.83 | 0.58 | 183 |
| ***Callianassa australis*** | 1.34 | 0.95 | 0 | 0 | 0.86 | 1.4 | 0.37 | 0.97 | 198 |
| ***Terebellides stroemi*** | 0 | 0 | 0 | 0 | 0.71 | 0 | 0 | 0 | 201 |
| ***Lumbrineris meteroana*** | 0 | 0 | 0 | 0 | 0.79 | 0 | 0 | 0 | 201 |
| ***Ampelisca anomala*** | 0 | 0 | 0 | 0 | 0.59 | 0.75 | 0 | 0 | 213 |
| ***Listriella lindae*** | 0 | 0.94 | 0 | 0 | 0.56 | 1.19 | 0 | 0.55 | 215 |
| ***Mediomastus capensis*** | 0 | 0 | 0 | 2.04 | 0.64 | 0 | 0.7 | 0.47 | 229 |
| ***Lumbrineris heteropoda difficilis*** | 0 | 0.95 | 0 | 0 | 0.67 | 0 | 0 | 0.89 | 246 |
| ***Tellina gilchristi*** | 0 | 0 | 0 | 0 | 0.38 | 0 | 0.51 | 0 | 256 |
| ***Ampelisca brevicornis*** | 0 | 0 | 0 | 0 | 0 | 0 | 0.36 | 0 | 297 |
| ***Centranthura caeca*** | 0 | 0 | 0 | 0 | 0 | 0 | 0.28 | 0 | 297 |
| ***Glycera convoluta*** | 0 | 0 | 0 | 0 | 0 | 0 | 0.31 | 0 | 297 |
| ***Urothoe grimaldi*** | 0 | 0 | 0 | 0 | 0 | 0 | 0.64 | 0 | 297 |
| ***Virgularia schultzei*** | 0 | 0 | 0 | 0 | 0 | 0 | 0.34 | 0 | 297 |
| ***Nephtys sphaerocirrata*** | 0 | 0 | 0 | 0 | 0.5 | 0 | 0.61 | 1.01 | 361 |
| ***Prionospio saldanha*** | 0 | 0 | 0 | 0 | 0 | 0 | 0.52 | 0.5 | 386 |
| ***Aricidea longobranchiata*** | 0 | 0 | 0 | 0 | 0 | 0 | 0 | 1.03 | 479 |
